# Delayed Presentation of a Traumatic Retroperitoneal Hematoma With Low-Grade Renal Injury After a Low-Speed Electric Scooter Handlebar Impact in a Child: A Case Report

**DOI:** 10.7759/cureus.118358

**Published:** 2026-10-22

**Authors:** Ibtihal Almeshawi, Ibrahim N Alghannam, Yara AlGoraini

**Affiliations:** 1 Pediatric Emergency Department, King Fahad Medical City, Riyadh, SAU

**Keywords:** blunt abdominal trauma, case report, electric scooter, handlebar injury, nonoperative management, pediatric emergency medicine, pediatric trauma, retroperitoneal hematoma

## Abstract

Electric scooters (e-scooters) are an increasingly common cause of injury in children, but their capacity to cause occult intra-abdominal injury is under-recognized. We report a seven-year-old boy with type 1 diabetes mellitus who presented to the pediatric emergency department seven days after a witnessed low-speed fall from an electric scooter with a direct handlebar impact to the right abdomen. He was initially asymptomatic, developed right-sided abdominal pain on the second day after injury, and became progressively pale. On arrival, he was hemodynamically stable, with right upper quadrant tenderness and fullness, no peritonism, and no external signs of injury. Bedside sonography showed no free intraperitoneal fluid, but marked anemia prompted contrast-enhanced computed tomography (CT), which demonstrated a large right retroperitoneal hematoma extending from the subhepatic region into the right hemipelvis, a right perinephric hematoma, and no active contrast extravasation. Targeted renal Doppler ultrasonography identified a small inferior-pole cortical laceration that was not discernible on computed tomography, and the renal injury was classified as low grade. He received a single red blood cell transfusion, was managed nonoperatively in a pediatric high-dependency unit, and was discharged in stable condition on day 5. This case shows that a low-speed handlebar impact without external signs can cause major retroperitoneal bleeding with a delayed presentation, that unexplained anemia after blunt abdominal trauma warrants cross-sectional imaging despite normal vital signs and negative bedside sonography, and that large retroperitoneal hematomas can be managed nonoperatively in stable children without active extravasation.

## Introduction

In the pediatric emergency department, the rise of electric scooters (e-scooters) as recreational vehicles has reshaped the trauma case mix in recent years. National emergency department surveillance data from the United States demonstrated a significant increase in pediatric e-scooter injuries between 2014 and 2023, with a 2.4-fold increase in the average annual number of injuries in the post-coronavirus disease (COVID) period (2021-2023) compared with the pre-COVID period (2014-2019) [1]. Most of these injuries are minor extremity or facial injuries; however, a smaller and far more concerning subset involves significant blunt abdominal trauma from handlebar impact [1,2]. The pediatric biomechanical setup is unfavorable. Adult-sized handlebars sit at the level of a child’s upper abdomen rather than the hip; the scooter platform places the child upright and leaning forward; helmets and protective equipment are inconsistently worn (in the most recent national pediatric series, helmet status was documented in only 19.4% of injury narratives, and 72.6% of these riders were not wearing a helmet) [1]; the rider can attain speeds well above what most caregivers expect. In a sudden braking or collision event, the child decelerates onto the handlebar, transmitting a focal compressive force directly into the abdomen; the same biomechanical principle has been described for bicycle handlebar injury [3].

Solid organ injury, most frequently to the liver, dominates the literature on pediatric handlebar-mechanism abdominal trauma [3]. Retroperitoneal hematomas are comparatively uncommon and, from the perspective of front-line emergency clinicians, far easier to miss. The retroperitoneal space can accommodate substantial volumes of blood before any change in heart rate or blood pressure becomes apparent. Symptoms may be delayed by several days, and the mechanism may sound trivial in the triage history (“just fell off the scooter”) [4]. The external examination may be unremarkable, with no bruising, seat-belt sign, or point of obvious tenderness, and the handlebar imprint frequently described in the bicycle literature is not consistently present [3]. Each of these features contributes to a diagnostic blind spot that is amplified in the e-scooter context.

We report the case of a seven-year-old boy who presented to our pediatric emergency department seven days after a witnessed low-speed fall from an e-scooter, with marked anemia and a large traumatic retroperitoneal hematoma that was successfully managed nonoperatively. We discuss the case from a pediatric emergency medicine perspective, focusing on the mechanism of injury and the reasons for delayed presentation, the cognitive pitfalls that the e-scooter mechanism creates for the front-line clinician, the role of cross-sectional imaging in an apparently low-acuity child after trauma, and the threshold for the nonoperative management of significant retroperitoneal injury.

## Case presentation

A seven-year-old boy with type 1 diabetes mellitus on a basal-bolus insulin regimen was brought to the pediatric emergency department by his parents with a five-day history of right-sided abdominal pain. Seven days earlier, while riding an e-scooter under family supervision, he had a witnessed low-speed fall in which he was thrown forward onto the handlebar, sustaining a direct blow to the right side of the abdomen. He was not wearing a helmet and did not lose consciousness. He walked away from the scene without visible injury and was not medically assessed at that time. He was initially asymptomatic but developed dull, non-radiating, right-sided abdominal pain on day 2 after injury, which gradually worsened over the subsequent 4-5 days. The progression of pain after an apparently trivial mechanism, together with increasing pallor and reduced oral intake, ultimately prompted presentation on day 7. There was no nausea, vomiting, fever, gross hematuria, or other urinary symptoms. There were no symptoms of a bleeding diathesis (easy bruising or epistaxis) and no family history of bleeding disorders.

His diabetes was well controlled, with no recent episodes of hypoglycemia. From a pediatric emergency medicine perspective, two elements of the history warranted attention at triage: a confirmed handlebar impact to the abdomen and a child who had become progressively pale and unwell rather than improving.

On arrival, his vital signs were as follows: heart rate, 103 beats/minute (within the upper range of normal for age); blood pressure, 109/59 mmHg; respiratory rate, 25 breaths/minute; oxygen saturation, 97% on room air; temperature, 36.8°C; capillary refill time, <2 seconds; and Glasgow Coma Scale score, 15. Point-of-care capillary blood glucose was 15 mmol/L. His weight was 24.4 kg (64th centile), and the Pediatric Trauma Score was 12. He appeared pale but well perfused. Abdominal examination revealed a soft abdomen with right upper quadrant tenderness and palpable fullness extending into the right flank, without rebound, guarding, or peritonism. Bowel sounds were present. There were no external signs of injury: no bruising, abrasions, seat-belt sign, or visible handlebar imprint. Genitourinary examination was unremarkable, and the remainder of the systemic examination was normal. The combination of pallor, a heart rate at the upper end of normal for age, and palpable abdominal fullness after a recent handlebar injury prompted parallel resuscitative and diagnostic workup despite an otherwise reassuring appearance.

Initial laboratory investigations demonstrated a hemoglobin level of 7.4 g/dL, white cell count of 7.83 × 10⁹/L, platelet count of 418 × 10⁹/L, a normal coagulation profile (prothrombin time, 14.3 seconds; international normalized ratio, 0.97; activated partial thromboplastin time, 27.5 seconds), serum creatinine of 35 µmol/L, lactate of 1.0 mmol/L, normal serum amylase and lipase, normal aminotransferases, and urinalysis without microscopic hematuria (Table 1). Blood was grouped and cross-matched for packed red cells.

**Table 1:** Laboratory investigations at presentation to the pediatric emergency department, with age-appropriate reference ranges.

Parameter	Patient value	Reference range	Unit
Hemoglobin	7.4	11.5-15.5	g/dL
White blood cell count	7.83	4.5-13.5	×10⁹/L
Platelet count	418	150-450	×10⁹/L
Prothrombin time	14.3	11.3-15.6	seconds
International normalized ratio	0.97	0.8-1.2	-
Activated partial thromboplastin time	27.5	24.5-37.5	seconds
Serum creatinine	35	27-62	µmol/L
Lactate	1.0	0.5-2.2	mmol/L
Capillary blood glucose (point of care)	15	3.9-7.8	mmol/L
Serum amylase	25	21-110	U/L
Serum lipase	20	13-60	U/L
Alanine aminotransferase	40	7-55	U/L
Aspartate aminotransferase	50	8-60	U/L
Urinalysis: red blood cells	0	0-5	per high-power field

Point-of-care ultrasound (focused assessment with sonography for trauma, FAST) performed in the resuscitation bay showed no free intraperitoneal fluid in the hepatorenal, splenorenal, or pelvic views (Figure 1). Because a negative FAST does not exclude significant injury, particularly retroperitoneal hemorrhage, and in view of the marked anemia and handlebar mechanism, contrast-enhanced computed tomography (CT) of the abdomen and pelvis was performed. CT revealed a large, complex, predominantly hypodense right subhepatic collection measuring 10.2 × 9 × 12.2 cm, extending along the right paracolic gutter into the right hemipelvis, anterior to the psoas muscle (Figures 2, 3). The heterogeneous internal components were consistent with an evolving hematoma, and there was no active contrast extravasation. A separate right perinephric hematoma measuring 4.6 × 3.7 cm was identified. The right kidney enhanced homogeneously, with no discernible cortical laceration or collecting-system injury on CT. There were no free intraperitoneal fluid and no hepatic, splenic, pancreatic, duodenal, or mesenteric injury.

**Figure 1:**
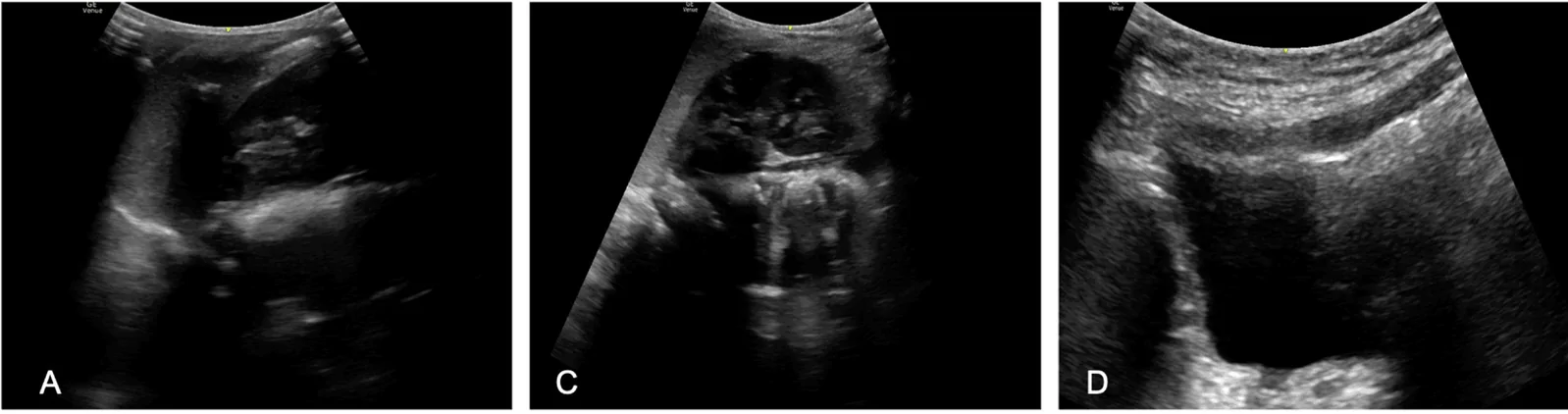
Point-of-care ultrasound (focused assessment with sonography for trauma) performed in the resuscitation bay. (A) Right upper quadrant (hepatorenal) view, (B) left upper quadrant (splenorenal) view, and (C) suprapubic view showing no free intraperitoneal fluid.

**Figure 2:**
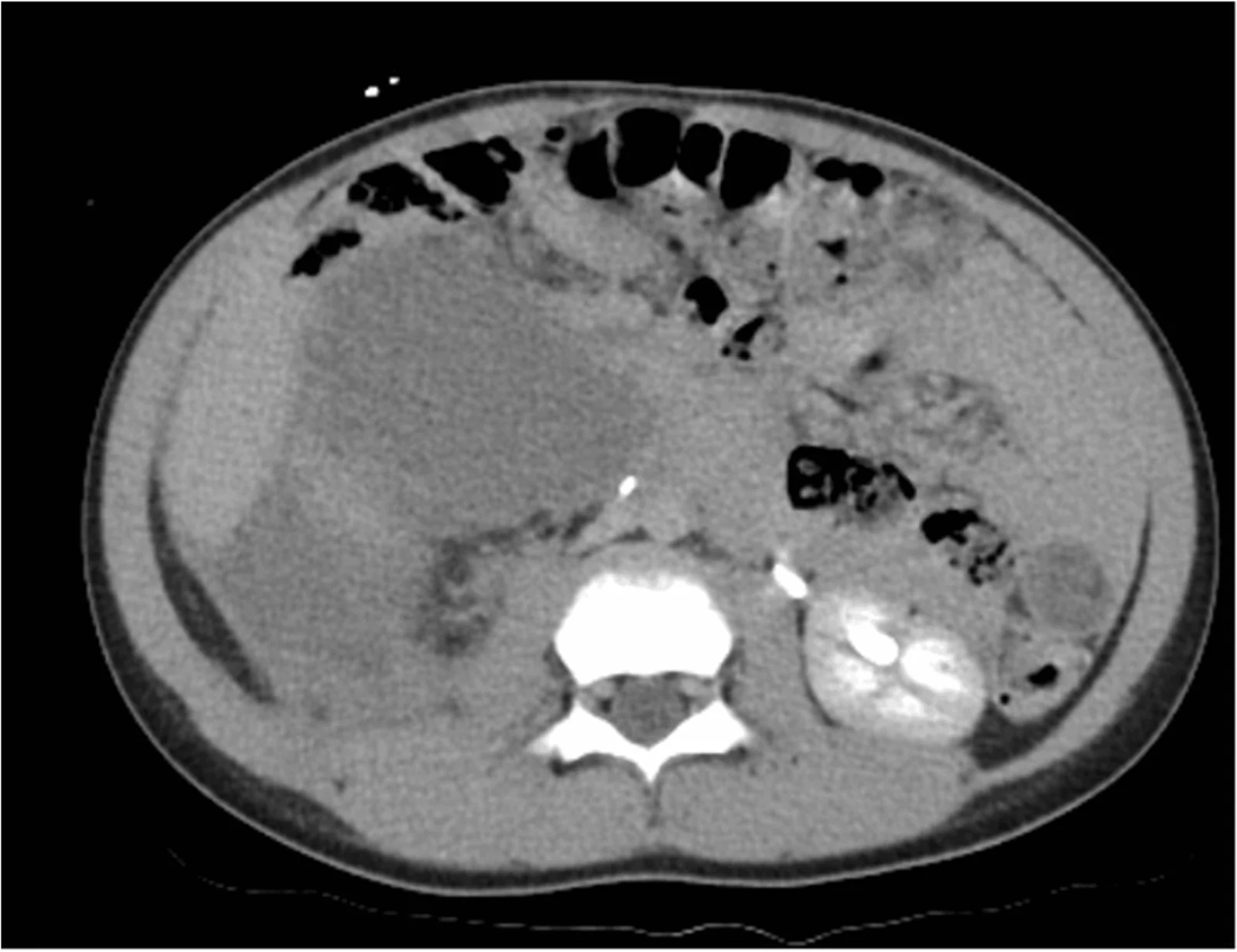
Contrast-enhanced computed tomography of the abdomen. Axial view at the level of the right kidney, demonstrating the retroperitoneal hematoma displacing adjacent structures anteriorly without the active extravasation of contrast.

**Figure 3:**
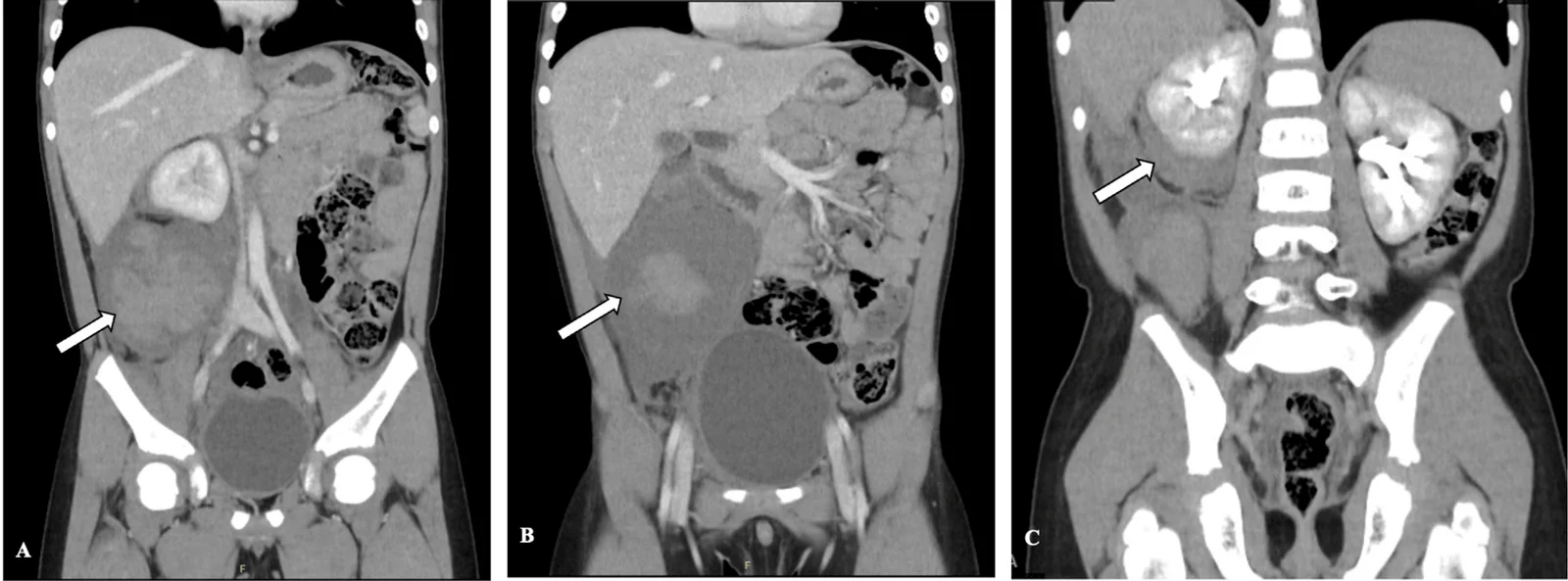
Contrast-enhanced computed tomography (CT) of the abdomen (coronal reformats). (A and B) Arrows indicate a large right retroperitoneal collection extending from the subhepatic region along the paracolic gutter into the right hemipelvis. (C) The arrow indicates the associated right perinephric hematoma. The right kidney shows homogeneous parenchymal enhancement.

Because the perinephric hematoma raised concern for an occult renal parenchymal injury, a targeted renal ultrasound with color and spectral Doppler was performed (Figures 4, 5). This showed a small, linear hypoechoic defect at the inferior pole of the right kidney, consistent with a superficial cortical laceration, with preserved arterial flow in the adjacent parenchyma, together with the known perinephric hematoma. The left kidney was normal.

**Figure 4:**
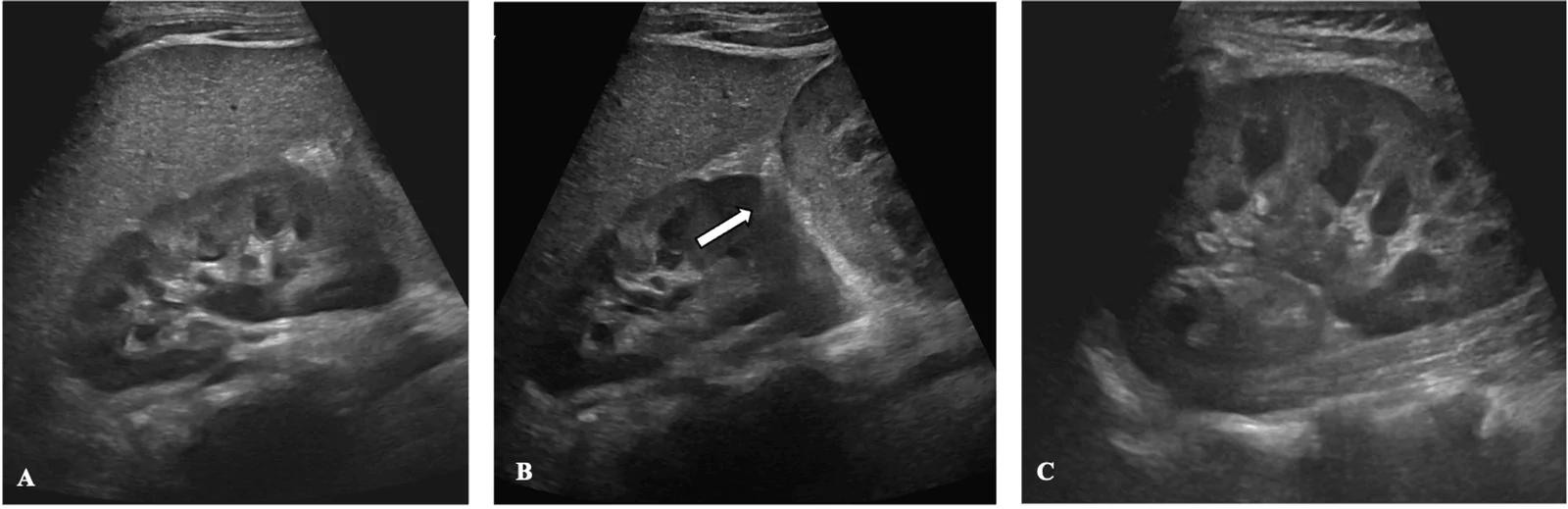
Renal ultrasound. (A) Grayscale image of the right kidney showing the inferior-pole region. (B) The arrow indicates a hypoechoic linear defect at the right inferior pole consistent with a cortical laceration; an adjacent perinephric collection is also demonstrated. (C) Grayscale image of the normal left kidney for comparison.

**Figure 5:**
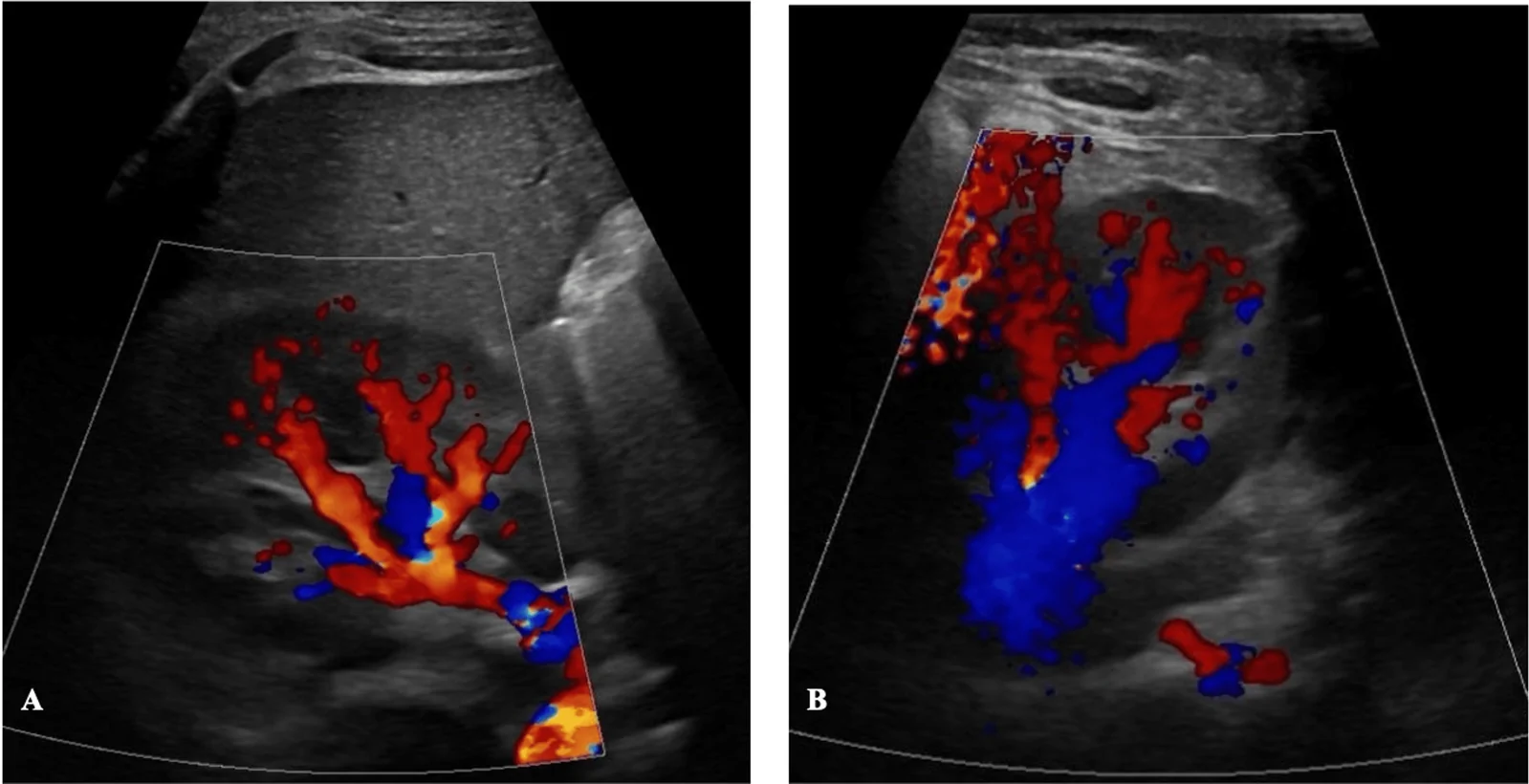
Color Doppler ultrasound of both kidneys. (A) Right kidney with preserved cortical and segmental arterial flow, including arterial flow adjacent to the inferior-pole laceration. (B) Normal left kidney for comparison.

The renal injury was graded using the 2018 American Association for the Surgery of Trauma (AAST) Organ Injury Scale [5]. Grading was performed retrospectively for this report, by consensus between the authors and pediatric radiology on the re-review of the original CT and ultrasound images. On CT alone, the perinephric hematoma confined to Gerota’s fascia meets the AAST grade II criterion, independent of any laceration; the absence of a visible laceration on CT is therefore not inconsistent with grade II. The superficial (≤1 cm) inferior-pole laceration subsequently identified on ultrasound, without urinary extravasation, also fulfils grade II criteria and did not change the grade. In the absence of active contrast extravasation, vascular injury, laceration deeper than 1 cm, or collecting-system involvement, criteria for grade III or higher were not met. The larger subhepatic-paracolic collection was graded as a retroperitoneal hematoma and is discussed separately below.

Within the pediatric emergency department, our initial priorities were resuscitation, risk stratification, and the assembly of a multidisciplinary team appropriate for significant abdominal injury in a child with type 1 diabetes mellitus. The patient was placed in the resuscitation bay for cardiorespiratory monitoring, two intravenous cannulas were inserted, he was kept nil by mouth, and weight-based intravenous maintenance fluids were started. Analgesia was provided with intravenous paracetamol and, as required, morphine, titrated to comfort while preserving the ability to perform serial abdominal examinations. His basal-bolus insulin regimen was continued, with capillary glucose checks every four hours in conjunction with the inpatient pediatric diabetes team. Tetanus immunization status was reviewed.

The pediatric surgery and urology teams were involved early. A joint review with pediatric radiology confirmed the injury pattern (a large retroperitoneal hematoma, a perinephric hematoma, and a superficial renal laceration) and the absence of active extravasation, mesenteric injury, or collecting-system disruption. The decision for nonoperative management was made jointly based on (1) hemodynamic stability with no clinical signs of ongoing bleeding, (2) the absence of active contrast extravasation on contrast-enhanced CT, (3) an injury pattern (retroperitoneal and perinephric hematoma with low-grade renal injury) for which contemporary pediatric trauma practice favors observation, and (4) the availability of a pediatric high-dependency setting with capacity for close monitoring and rapid surgical escalation if needed.

The patient received one weight-appropriate transfusion of packed red blood cells (10-15 mL/kg, per institutional pediatric guidance), with hemoglobin increasing from 7.4 g/dL to 10 g/dL after transfusion. Transfusion was driven less by the absolute hemoglobin value than by the combination of substantial recent blood loss, the size of the collection, and the planned nonoperative pathway.

Conservative management consisted of admission to the pediatric high-dependency unit with cardiorespiratory monitoring; serial hemoglobin measurements every six hours initially, subsequently de-escalated as the hemoglobin trend stabilized; serial abdominal examinations by the pediatric surgical and emergency medicine teams; strict bed rest with graded mobilization; structured multidisciplinary review by pediatric surgery, urology, and the diabetes team; explicit pre-discharge counseling on activity restriction (no contact sport, scooter, or bicycle) and warning signs that should trigger return to the emergency department; and planned outpatient follow-up.

The patient’s condition remained stable during admission. Hemoglobin levels stabilized after the single transfusion, abdominal pain settled progressively, glycemic control remained at baseline under the diabetes team, and the patient tolerated graded mobilization without symptom recurrence. The patient did not require surgical or interventional radiological intervention. The patient was discharged on day 5 with a structured outpatient follow-up, laboratory tests, and repeat ultrasonography.

## Discussion

From the standpoint of pediatric emergency medicine, four features of this case deserve emphasis: the mechanism of injury and its relationship to the delayed presentation, the deceptive nature of the e-scooter mechanism, the role of unexplained anemia as a diagnostic trigger, and the criteria for the safe nonoperative management of significant retroperitoneal injury in a child.

Electric scooter as a pediatric abdominal trauma vector

E-scooter injuries are an established and growing component of the pediatric emergency caseload. National surveillance data from the United States documented a significant increase in pediatric e-scooter injuries from 2014 to 2023, with a 2.4-fold increase in the post-COVID period; falls from the scooter accounted for 69.1% of injuries, and most riders whose helmet status was recorded were unhelmeted [1]. A single-center pediatric series mirrors these patterns and confirms that fractures, head injuries, and dental injuries dominate the case mix [2]. The proportion of e-scooter incidents resulting in significant abdominal trauma is small but not negligible, and the abdominal injuries that do occur tend to share a recognizable pattern.

Mechanism of injury and delayed presentation

The handlebar of an adult- or near-adult-sized e-scooter sits at the upper abdominal height of a 5-10-year-old child. In sudden braking, a curb strike, or a forward fall, the child decelerates onto the handlebar end, which concentrates the impact force over a small contact area and compresses the abdominal contents against the vertebral column and posterior abdominal wall. Finite-element simulations in a six-year-old child body model have shown that the shape and rigidity of the handlebar end strongly influence abdominal organ loading, with standard and damaged handlebar ends producing the highest injury risk and child-specific grips reducing it [6]. Such focal compression and shear can injure retroperitoneal structures, including the kidney, duodenum, pancreas, mesenteric root, and retroperitoneal vessels, while leaving the skin intact [3]. In our patient, the right-sided impact, the right perinephric hematoma, and the inferior-pole laceration point to the right kidney and perirenal tissues as the most likely bleeding source. However, the origin of the larger subhepatic-paracolic component could not be established with certainty, and bleeding from small retroperitoneal vessels cannot be excluded. We emphasize that the hematoma was not shown to have formed late. The retroperitoneum can accommodate a large volume of blood, and low-pressure bleeding is partly self-tamponaded, so symptoms may evolve gradually as the hematoma enlarges, stretches the surrounding tissues, and produces anemia [4]. The heterogeneous, evolving appearance of the collection on CT is consistent with blood that had accumulated over several days. We therefore interpret this case as a delayed presentation and diagnosis of a hematoma that most likely began at or soon after the time of injury, rather than as delayed hematoma formation; in the absence of imaging at the time of injury, the precise timing of bleeding cannot be determined.

This is the same biomechanical principle described for bicycle-related handlebar injuries. A systematic review of 1,072 children with bicycle handlebar thoracoabdominal injuries identified the liver as the most frequently injured organ; notably, 2.5% of children were initially discharged and returned with worsening symptoms, 85.2% of whom required surgery [3]. In the e-scooter context, three pediatric-specific aggravators compound the risk: higher attainable speeds than a child can achieve under their own power on a bicycle, a forward-leaning rider posture that places the handlebar more directly in line with the upper abdomen, and inconsistent helmet and protective equipment use [1,3]. A recent pediatric report described a 12-year-old boy with a retroperitoneal hematoma after a bicycle handlebar injury, in whom operative exploration revealed mesenteric and vascular injury [7]. Another described a retroperitoneal hematoma secondary to traumatic liver injury in an infant, in whom the atypical bleeding pattern delayed the appreciation of injury severity [8]. These reports support the principle that handlebar-mechanism abdominal trauma in children may present atypically, and our case extends this principle to modern e-scooters.

Why is the e-scooter mechanism a diagnostic trap?

In our department, the cognitive risk associated with the e-scooter abdominal mechanism lies in the mismatch between the apparent severity at the scene and the energy actually transferred. The child often walks away, with no damaged helmet, twisted metal, or obvious deformity, and the triage history is typically benign (“just fell off the scooter”). When the child presents days later with vague abdominal pain, the original mechanism is easily discounted, especially in a busy department operating under a high cognitive load. A similar phenomenon is seen in the bicycle handlebar literature, in which a small but clinically important fraction of children are initially discharged and return with worsening symptoms, most of whom require surgery [3]. In the present case, the patient was initially asymptomatic, developed pain only on day 2, and presented seven days after injury. The delayed onset of symptoms is a recognized feature of retroperitoneal bleeding [4]. Pediatric emergency clinicians should regard any handlebar-mechanism scooter incident as a potential abdominal injury until proven otherwise, even when the rest of the picture is reassuring.

Anemia as the diagnostic pivot

In our case, the most decisive trigger for cross-sectional imaging was not the vital signs, the abdominal examination, or the mechanism in isolation but the degree of unexplained anemia. In pediatric blunt trauma, retroperitoneal blood loss may be substantial before the heart rate and blood pressure move outside normal limits, because children maintain physiological compensation until late. In our practice, a hemoglobin level of 7.4 g/dL in a child after blunt abdominal trauma, without a known cause of chronic anemia, should prompt cross-sectional imaging even when the child appears well. Clinical decision rules offer only partial support in this scenario. The Pediatric Emergency Care Applied Research Network (PECARN) intra-abdominal injury rule comprises seven history and examination variables (the evidence of abdominal wall trauma or seat-belt sign, Glasgow Coma Scale score of <14, abdominal tenderness, thoracic wall trauma, the complaint of abdominal pain, decreased breath sounds, and vomiting) and, in a multicenter prospective validation study of 7,542 children, identified those at a very low risk of intra-abdominal injury requiring acute intervention with a sensitivity of 100% [9]. Laboratory values, including hemoglobin, are not part of the rule, and children injured more than 24 hours before presentation were excluded from the validation cohort; to our knowledge, no published subgroup analysis addresses children presenting days after injury. Our patient, who presented on day 7, was therefore outside the validated population, and the rule cannot formally be applied to him. We note only that he had two of the rule’s predictor variables (abdominal pain and abdominal tenderness), which suggests that he would probably not have been classified as very low risk had he presented acutely; this observation is illustrative and should not be interpreted as evidence for the rule’s performance in delayed presentations. Urinalysis and the Pediatric Trauma Score are useful adjuncts but should not delay imaging when anemia is unexplained.

Imaging strategy in the stable child after trauma

Abdominopelvic contrast-enhanced CT remains the diagnostic standard for hemodynamically stable children with blunt abdominal trauma [10]. FAST is useful but limited: In a prospective multi-institutional study of 2,188 children, its sensitivity was only 27.8% for intra-abdominal injury and 44.4% for intra-abdominal injury requiring intervention, and it is inherently insensitive to retroperitoneal hemorrhage, in which the absence of intraperitoneal fluid may falsely reassure [11]. In our case, FAST was negative despite a large retroperitoneal hematoma. Ultrasound is more useful as a complement to CT than as a substitute, particularly for characterizing the renal parenchyma. The small inferior-pole laceration in our patient was identified on targeted Doppler ultrasound but was not discernible on the initial CT. We do not interpret this as a CT miss. On retrospective re-review of the CT images with pediatric radiology, no definite parenchymal defect could be identified, and the lesion was most likely below the effective resolution of CT, owing to its small size and superficial location, partial-volume averaging, and the adjacent perinephric hematoma obscuring the cortical margin. High-frequency ultrasound in a slim child provides a higher spatial resolution of the superficial renal cortex. Importantly, the sonographic finding did not change the AAST grade or management, but it did clarify the likely source of the perinephric bleeding, illustrating the value of multimodal assessment in pediatric renal injury. Contemporary pediatric guidance, including the Arizona-Texas-Oklahoma-Memphis-Arkansas Consortium (ATOMAC) pathway, emphasizes judicious CT use to balance diagnostic yield against ionizing radiation exposure in children [10]; we apply this principle through case-by-case discussion with pediatric radiology rather than a rigid algorithm.

Nonoperative management criteria

The management of this case followed the principles of contemporary pediatric trauma care. The ATOMAC pathway for pediatric blunt solid organ injury, the dominant evidence-based framework of the last decade, supports nonoperative management in hemodynamically stable children, with monitoring intensity stratified by physiological status rather than by a fixed length of stay [10]. The 2019 World Society of Emergency Surgery (WSES)-AAST kidney and urotrauma guidelines extend this principle to renal injury and recommend nonoperative management in hemodynamically stable patients in the absence of other indications for surgery [12]. In our department, the criteria for the nonoperative management of significant retroperitoneal or renal injury include hemodynamic stability after initial resuscitation, the absence of active contrast extravasation, the absence of associated injuries mandating operative exploration, access to a pediatric high-dependency setting with capacity for serial monitoring, and a clear escalation pathway with pediatric surgery and interventional radiology if the trajectory changes. Our patient met all these criteria and was successfully managed nonoperatively.

Limitations

This report describes a single patient, and its findings cannot be generalized. Repeat cross-sectional imaging was not performed during admission. The AAST grade was assigned retrospectively, in conjunction with pediatric radiology. The source of the larger retroperitoneal component could not be determined with certainty, and without imaging at the time of injury, the timing of hematoma formation cannot be established. Follow-up was limited to three weeks. The contribution of type 1 diabetes mellitus to the delayed presentation or recovery cannot be established from this report.

## Conclusions

A low-speed e-scooter handlebar impact without external signs of injury can cause a large retroperitoneal hematoma in a child, and symptoms may evolve over days, delaying presentation and diagnosis. Unexplained anemia after blunt abdominal trauma should prompt cross-sectional imaging, even when vital signs are normal and FAST is negative. In stable children without active extravasation, a large retroperitoneal hematoma with low-grade renal injury can be managed nonoperatively with high-dependency monitoring, serial hemoglobin measurements and abdominal examinations, multidisciplinary input, and a clear escalation plan.
